# Tree-Guided Transformer for Sensor-Based Ecological Image Feature Extraction and Multitarget Recognition in Agricultural Systems

**DOI:** 10.3390/s25196206

**Published:** 2025-10-07

**Authors:** Yiqiang Sun, Zigang Huang, Linfeng Yang, Zihuan Wang, Mingzhuo Ruan, Jingchao Suo, Shuo Yan

**Affiliations:** 1 China Agricultural University, Beijing 100083, China; 2 National School of Development, Peking University, Beijing 100871, China; 3 Tsinghua University, Beijing 100084, China; 4 State Key Laboratory of Agricultural and Forestry Biosecurity, China Agricultural University, Beijing 100083, China

**Keywords:** object detection in sensor systems, sensors-based image feature extraction, multimodal agricultural data, semantic alignment via prompting, pest–predator recognition

## Abstract

Farmland ecosystems present complex pest–predator co-occurrence patterns, posing significant challenges for image-based multitarget recognition and ecological modeling in sensor-driven computer vision tasks. To address these issues, this study introduces a tree-guided Transformer framework enhanced with a knowledge-augmented co-attention mechanism, enabling effective feature extraction from sensor-acquired images. A hierarchical ecological taxonomy (Phylum–Family Species) guides prompt-driven semantic reasoning, while an ecological knowledge graph enriches visual representations by embedding co-occurrence priors. A multimodal dataset containing 60 pest and predator categories with annotated images and semantic descriptions was constructed for evaluation. Experimental results demonstrate that the proposed method achieves 90.4% precision, 86.7% recall, and 88.5% F1-score in image classification, along with 82.3% hierarchical accuracy. In detection tasks, it attains 91.6% precision and 86.3% mAP@50, with 80.5% co-occurrence accuracy. For hierarchical reasoning and knowledge-enhanced tasks, F1-scores reach 88.5% and 89.7%, respectively. These results highlight the framework’s strong capability in extracting structured, semantically aligned image features under real-world sensor conditions, offering an interpretable and generalizable approach for intelligent agricultural monitoring.

## 1. Introduction

In recent years, with the rise in smart agriculture and green pest control, computer vision and AI-based crop pest monitoring and ecological management have become key directions in agricultural informatization research [1]. Pests and diseases are major constraints to sustainable agriculture, directly affecting crop yields and ecosystem stability [2]. In agroecosystems, complex interactions—such as predation, parasitism, competition, and symbiosis—exist between pests and their natural enemies, shaping both individual-level outbreak dynamics and system-level control strategies [3]. Thus, developing intelligent models that can recognize multiple organisms and understand their ecological co-occurrence is crucial for precision agriculture and effective biological control.

Deep learning has been widely applied in agricultural object detection and classification, significantly improving pest monitoring automation [4]. The YOLO series, known for its end-to-end structure and efficiency, has gained attention in agricultural vision tasks. For instance, Yang et al. proposed YOLOv5s-pest, achieving 92.5% mAP@0.5 on the IP16 dataset and enhancing small pest detection [5]. Wang et al. developed Insect-YOLO for low-resolution field images, reaching 93.8% mAP@0.5 on 8–12MP images [6]. Vision Transformers (ViT), known for strong global modeling, have also been introduced. Xie et al. proposed a multi-scale ViT (SFA-ViT) that performed well on IP102 and Plant Village datasets [7], while Jamali et al. combined CNN and ViT to capture both local and global features, achieving 78.15% accuracy on a custom dataset [8]. These models show strong performance under standard conditions and have advanced intelligent agricultural sensing. However, challenges remain in model robustness and ecological interpretability under complex field conditions. Farmland ecosystems include diverse pests and natural enemies with similar appearances and variable scales, often blending into noisy backgrounds such as leaves or soil, leading to false positives/negatives [9,10]. Moreover, these organisms often interact ecologically through predation, inhibition, or commensalism—but most current models focus on individual recognition, lacking the ability to model interspecies relationships and ecological hierarchies [11]. This “recognition without understanding” limits ecological insight and restricts support for green pest control strategies.

To address these issues, we propose an intelligent pest–natural enemy recognition framework that integrates hierarchical reasoning and knowledge-enhanced visual modeling. A multimodal collaboration mechanism is designed to improve recognition accuracy and ecological interpretability in complex farmland environments. The main contributions of this study are as follows:

1. A pest–natural enemy recognition method guided by hierarchical structure is proposed, wherein an ecological taxonomy tree and tree-guided prompts are designed to facilitate hierarchical inference through a large language model, enhancing the discriminability of rare and confusing classes.
2. A knowledge-enhanced visual recognition backbone is developed by integrating ecological knowledge graphs into a ViT architecture and introducing a co-occurrence attention mechanism, enabling improved modeling of semantic relationships and spatial structures between pests and natural enemies.
3. A multimodal dataset with ecological semantic alignment is also constructed, comprising images, texts, and hierarchical labels, with expert-annotated co-occurrence relationships to support semantic learning and ecological reasoning.
4. The proposed method is thoroughly validated across multiple tasks. Comparative and ablation experiments demonstrate superior performance in fine-grained recognition, ecological inference, and structural consistency, highlighting its potential for scalable applications in intelligent agriculture.

## 2. Related Work

### 2.1. Insect Recognition and Predator Modeling

Recent advances in object detection and classification have significantly improved agricultural pest recognition. Two-stage models like Faster R-CNN achieve over 85% accuracy in detecting small pests such as aphids and red spiders in complex farmland scenes [12]. One-stage models, particularly the YOLO series (e.g., YOLOv5, YOLOv8), excel in real-time applications; for example, YOLO-based systems achieve up to 30 fps for monitoring cotton bollworms in greenhouses [13]. Vision Transformers (ViT), which divide images into patch sequences and use self-attention to capture global features, outperform convolutional networks (e.g., ResNet) in distinguishing visually similar pests like Ostrinia furnacalis and Cnaphalocrocis medinalis. ViT achieves 10–15% higher mAP and up to 95.72% classification accuracy without data augmentation [14,15]. In contrast, predator recognition and pest–predator relationship modeling are still underdeveloped. Most studies focus on individual species classification. For instance, SVMs combined with optimized feature extraction have improved the identification of beneficial insects like lady beetles to around 80% accuracy [16], but often ignore ecological interactions [17].

Traditional ecological models—such as Lotka–Volterra equations and niche models—have been used to simulate predator–prey dynamics, feedback mechanisms, and resource regulation. Tonnang et al. applied Lotka–Volterra equations to Plutella xylostella populations on cabbage, accurately predicting pest density per plant in alignment with field data [18]. AI methods have also been explored for ecological modeling. Rouabah et al. evaluated linear models, regression trees, and random forests to predict aphid abundance, parasitism rates, and predator populations (lady beetles, hoverflies, and lacewings) in cereal systems, highlighting the strength of AI in modeling pest–predator interactions [19]. These approaches support early warnings, capture delayed predator responses, and help define local control thresholds, providing a basis for precision pest management.

### 2.2. Knowledge-Augmented Visual Understanding and Co-Occurrence Modeling

Vision Transformers (ViTs) enhance multi-object understanding by capturing spatial and semantic relationships, outperforming CNNs in tasks like pest–predator co-occurrence [14,15]. Swin Transformer improves efficiency with windowed attention, while DeiT accelerates detection via knowledge distillation for edge devices. These models infer not just object presence but also spatial relations (e.g., “lady beetle near aphids”). Knowledge graphs (KGs) embed ecological relationships into visual models, aiding semantic differentiation (e.g., predators vs. herbivores). However, most approaches overlook broader co-occurrence patterns in ecological contexts. To address this, we introduce a co-occurrence attention mechanism that incorporates ecological priors into transformer attention, guiding the model toward relevant object pairs and improving relationship inference.

## 3. Materials and Method

### 3.1. Data Collection

**Figure 1:**
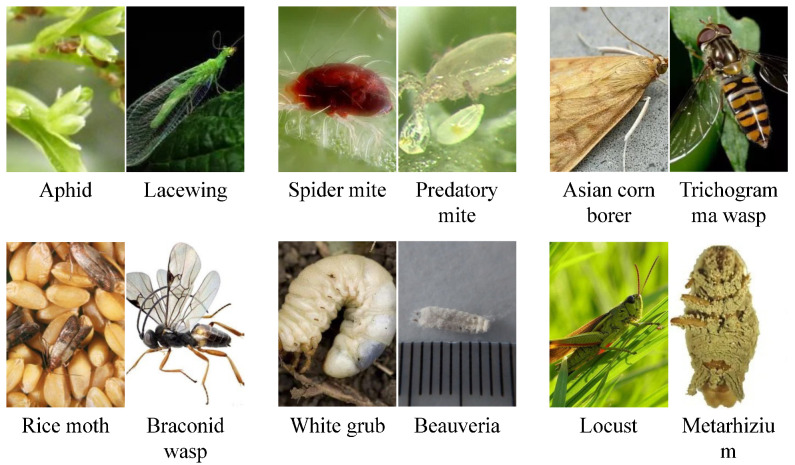
Image data samples.

In this study, a multimodal pest–predator co-occurrence dataset was constructed to facilitate species identification and ecological relationship modeling within agricultural ecosystem images, as shown in Table 1. The dataset encompasses image data, ecological semantic descriptions, and structured hierarchical labels. The majority of image data were obtained through in-field collection, complemented by samples from agricultural databases and the internet. Image acquisition employed dual-modality imaging devices, including visible light (RGB) cameras and infrared thermal imagers, with resolutions of 1920 × 1080 and 640 × 480, respectively, as shown in Figure 1. The collection spanned from 2023 to 2025, covering spring sowing, summer growth, and early autumn harvest stages to ensure comprehensive ecological seasonality.

During data acquisition, representative plant regions were selected as primary targets, and environmental parameters such as illumination intensity and wind speed were concurrently recorded to supplement labeling. The infrared channel was specifically leveraged to enhance detection of camouflaged targets and objects within complex backgrounds, thereby improving the robustness of visual models under occlusion and variable lighting conditions.

Ecological textual descriptions were sourced from multiple channels, including entomological atlases published by the Chinese Academy of Agricultural Sciences and open-access agricultural biodiversity databases such as FAO and GBIF. The collected descriptions underwent preprocessing procedures, including denoising, normalization, and entity standardization. Taxonomic information, such as morphological traits, behavioral patterns, and ecological niches, was extracted and aligned with corresponding image targets to support downstream KG embedding.

Label annotation was performed in a two-stage process. Initially, visually distinguishable individuals within each image were annotated with multi-class bounding boxes, and species-level labels were manually assigned. Subsequently, based on expert ecological knowledge, pest–predator co-occurrence relationships—including predation, competition, and cohabitation—were identified and structurally recorded as entity pairs with associated relational attributes. The hierarchical taxonomy was defined across three levels (order–family-species), comprising 40 common agricultural pests and 20 types of natural predators, covering multiple taxonomic groups such as Lepidoptera, Hemiptera, Hymenoptera, and Coleoptera. The resulting dataset integrates image, textual, and structural label modalities, enabling multimodal, multi-task, and hierarchical modeling, and provides a solid foundation for pest–predator co-occurrence recognition and ecological modeling in this study.

### 3.2. Data Augmentation

In farmland pest, disease, and predator recognition tasks, raw data acquisition is often subject to multiple influences from environmental complexity and varying collection conditions. As a result, the collected images commonly exhibit uneven illumination, background interference, target occlusion, and morphological variations. Simultaneously, ecological textual descriptions may contain spelling errors, redundant symbols, and inconsistent species naming. These types of noise and inconsistencies not only reduce model recognition accuracy in real-world scenarios but also weaken generalization performance and the stability of ecological relationship modeling. Therefore, systematic preprocessing and augmentation must be conducted prior to model training, aiming to restore and expand the diversity of real-world conditions while providing clean and structured inputs for subsequent vision–semantic joint modeling.

To simulate common phenomena in practical farmland environments, such as illumination changes, target occlusion, and morphological diversity, various image augmentation strategies were adopted, as shown in Algorithm 1, including background perturbation, occlusion simulation, and local deformation. Specifically, background perturbation was achieved by randomly altering the brightness, contrast, and saturation of background regions to mimic variations in soil, foliage, and sky across different temporal and climatic conditions [20]:

(1) $$\hat{I}\left(x,y\right)=\left\{\begin{array}{cc} \alpha \cdot I(x,y)+\beta , & \mathrm{if}\;\left(x,y\right)\in \Omega_{\mathrm{bg}}\\ I(x,y), & \mathrm{otherwise} \end{array}\right..$$

Here, I(x,y) and $\hat{I}\left(x,y\right)$ denote the original and processed image pixels, respectively, $\Omega_{\mathrm{bg}}$ represents the background pixel set, and α∈[0.7,1.3] and β∈[−20,20] are brightness scaling and offset coefficients. This operation was designed to reduce the risk of overfitting to specific background textures or illumination patterns, thereby improving generalization across diverse farmland settings.

Occlusion simulation was performed by generating rectangular or irregular masking regions at random locations within the image to partially obscure target objects. This process mimics occlusions caused by foliage, soil, or other elements and encourages the model to learn discriminative local features rather than relying on complete contours [21]:

(2) $$\hat{I}\left(x,y\right)=\left\{\begin{array}{cc} m, & \mathrm{if}\;\left(x,y\right)\in \Omega_{\mathrm{occ}}\\ I(x,y), & \mathrm{otherwise} \end{array}\right..$$

Here, *m* is a random color value for the occlusion region, and $\Omega_{\mathrm{occ}}$ denotes the occluded pixel set. Local deformation was applied to enhance the model’s robustness to variations in insect posture, scale, and deformation. Affine or elastic transformations were applied to local image regions to simulate natural variations in insect orientations and angles [22]:

(3) $$\left[\begin{array}{c} x^{\prime }\;y^{\prime } \end{array}\right]=A\left[\begin{array}{c} x\;y \end{array}\right]+t.$$

Here, $A\in R^{2\times 2}$ is the affine transformation matrix, and $t\in R^{2}$ is the translation vector.

For textual data, field-collected ecological descriptions frequently contain spelling errors, redundant symbols, and multiple naming conventions for the same species. Direct usage of such text introduces misalignment with knowledge representations, thereby impairing subsequent semantic reasoning. To address this issue, a denoising step was first applied to remove irrelevant characters, repeated punctuation, and non-semantic markers [23]:

(4) $$T^{\prime }=R_{\mathrm{noise}}\left(T\right),$$

where *T* denotes the original ecological text, $T^{\prime }$ is the cleaned, standardized version, and $R_{\mathrm{noise}}$ represents a rule-based text cleaning function. Next, entity normalization was performed by mapping synonyms and colloquial names to standardized scientific names (Latin binomials), ensuring alignment with entities in the KG [24]:

(5) $$e_{\mathrm{std}}=\phi \left(e_{\mathrm{raw}}\right).$$

Here, $e_{\mathrm{raw}}$ represents the species name in the original text, $e_{\mathrm{std}}$ is the mapped scientific name, and ϕ(·) denotes a taxonomy-based normalization function based on standard biological dictionaries such as GBIF The whole process is shown in Algorithm 2.
**Algorithm 1** Image Augmentation
 **Require:** 
Image *I*, background mask $\Omega_{\mathrm{bg}}$, parameters $\left\{p_{bg},\alpha ,\beta ,p_{occ},shape,size,p_{affine},A,t\right\}$
 **Ensure:** 
Augmented image $\hat{I}$  1:$\hat{I}\leftarrow I$  2:**if** RAND() $<p_{bg}$ **then**  3:     Sample α,β  4:     **for all** pixel (x,y) in *I* **do**  5:           **if** $\left(x,y\right)\in \Omega_{\mathrm{bg}}$ **then**  6:              $\hat{I}\left(x,y\right)\leftarrow \mathrm{CLIP}\left(\alpha \cdot I\left(x,y\right)+\beta \right)$  7:           **else**  8:              $\hat{I}\left(x,y\right)\leftarrow I\left(x,y\right)$  9:           **end if**10:     **end for**11:**end if**12:**if** RAND() $<p_{occ}$ **then**13:     S← SAMPLE_SHAPE(shape, size)14:     m← RANDOM_COLOR()15:     **for all** (x,y)∈S **do**16:          $\hat{I}\left(x,y\right)\leftarrow m$17:     **end for**18:**end if**19:**if** RAND() $<p_{affine}$ **then**20:      Sample A,t21:      **for all** region $R\subset \hat{I}$ **do**22:            **for all** (x,y)∈R **do**23:               ${\left[x^{\prime },y^{\prime }\right]}^{T}\leftarrow A{\left[x,y\right]}^{T}+t$24:               $\hat{I}\left(x^{\prime },y^{\prime }\right)\leftarrow \mathrm{INTERP}\left(\hat{I}\left(x,y\right)\right)$25:            **end for**26:     **end for**27:**end if**28:**return** $\hat{I}$

In the labeling process, a pest–predator ecological taxonomy tree was constructed to convert manually annotated flat labels into hierarchical labels and generate co-occurrence pairs. For each species *c*, its complete path from phylum to species was retrieved from the taxonomy tree T [25]:

(6) $$L\left(c\right)=l_{1},l_{2},\ldots ,l_{k},\;c\in l_{k}.$$

Here, L(c) is the set of hierarchical labels for species *c*, with $l_{1},l_{2},\ldots ,l_{k}$ representing classification levels such as phylum, class, order, family, genus, and species. The taxonomy tree T defines these relationships, where *c* corresponds to a leaf node.

Within each image, when both pest set P and predator set E are present, all possible ordered pairs were constructed [11]:

(7) C=(p,e),|,p∈P,e∈E.

Here, P and E denote the sets of pest and predator species within the image, and each (p,e) represents a co-occurrence relation between a pest *p* and a predator *e*. The set C contains all candidate pest–predator pairs and was used to supervise model learning of ecological co-occurrence patterns. These pairs not only provide direct supervision for relationship modeling but also serve as structured priors for ecological semantic reasoning. Through the aforementioned preprocessing and augmentation procedures, original information was maximally preserved while enhancing data diversity and consistency, laying a solid foundation for model robustness in real-world agricultural environments.
**Algorithm 2** Text Preprocessing and Entity Normalization
 **Require:** 
Raw text *T*, synonym dictionary dicts
 **Ensure:** 
Cleaned text $T^{\prime }$, standardized entities $E_{std}$  1:$T^{\prime }\leftarrow R_{noise}\left(T\right)$                     ▹ rule-based cleaning  2:$E_{raw}\leftarrow$ NER_EXTRACT($T^{\prime }$)  3:$E_{std}\leftarrow \emptyset$  4:**for all**$\;e_{raw}\in E_{raw}\;$**do**  5:      $e_{std}\leftarrow \phi \left(e_{raw},\mathrm{dicts}\right)$  6:      **if** $e_{std}\neq$ NULL **then**  7:          $E_{std}\leftarrow E_{std}\cup \left\{e_{std}\right\}$  8:      **end if**  9:**end for**10:**return**$\;T^{\prime },E_{std}$

### 3.3. Proposed Method

#### 3.3.1. Overall

**Figure 2:**
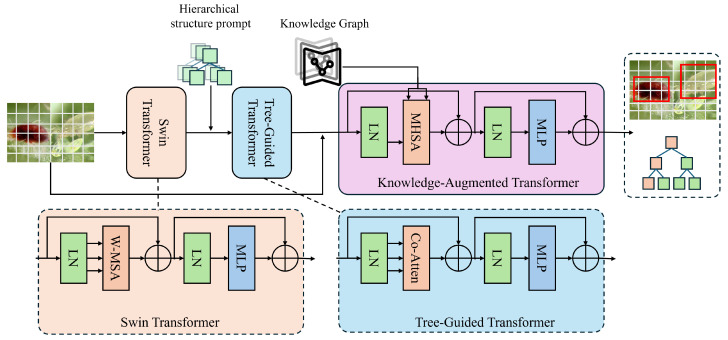
Overall framework of the proposed tree-guided transformer for sensor-based ecological image feature extraction and multi-target recognition.

The proposed pest–predator hierarchical recognition and co-occurrence modeling framework, as shown in Figure 2, has been constructed as a multi-module collaborative deep learning system, designed to address ecological multi-object recognition and semantic reasoning tasks in agricultural field images. The input to the model consists of preprocessed images and corresponding structured label information. A Swin Transformer-based visual backbone is employed to extract multi-scale image features by modeling both spatial and semantic relationships through a hierarchical windowed self-attention mechanism, producing high-dimensional regional feature representations. Subsequently, detected object regions are processed via RoI alignment to extract local feature vectors, which are then embedded and forwarded to the Tree-guided Transformer module. This module incorporates a pre-defined three-level ecological taxonomy of pests and predators to construct hierarchical prompts, guiding the large language model to generate class predictions step by step, enabling structured recognition from order to species with enhanced generalizability and interpretability.

To further improve the model’s capacity to perceive ecological relationships among targets, a knowledge-augmented transformer module is introduced. This component integrates semantic embeddings of entity nodes from an agricultural KG into the attention layers of the visual backbone. These embeddings are fused with the transformer’s multi-head attention computations, enabling the model to capture ecological semantic patterns such as predation, cohabitation, and competition under multi-object coexistence conditions. A co-occurrence attention mechanism is designed within the attention layers, incorporating structural relational priors between entities from the KG into the attention weighting process, thereby enhancing the model’s ability to represent semantic dependencies among objects.

The model produces two main outputs: the hierarchical classification path for each detected target and the predicted ecological co-occurrence relationships between object pairs. These are jointly optimized through a multi-task loss function that integrates classification accuracy, structural path consistency, and ecological relationship reasoning. This design ensures that the model not only achieves high recognition performance but also exhibits structural and relational interpretability, facilitating a transition from visual recognition to ecological understanding [26].

#### 3.3.2. Tree-Guided Transformer

The Tree-guided Transformer module is designed to enable Transformer structure to perform structured and hierarchy-aware semantic reasoning for object recognition. In pest–predator co-occurrence imagery, conventional classification models often adopt a flat label structure, disregarding the intrinsic semantic hierarchy among biological entities. To address this limitation, a three-level ecological taxonomy tree—spanning order, family, and species—has been integrated into the Tree-Guided Prompt design. This structure supports step-wise semantic generation, combined with multi-step decoding, shareable cache optimization, and hierarchical consistency constraints, forming a complete hierarchical reasoning pipeline.

**Figure 3:**
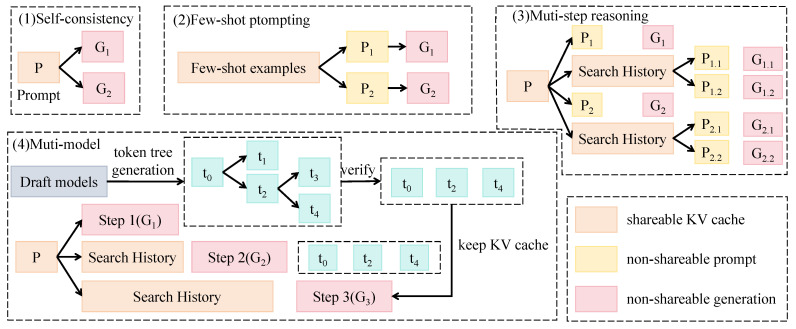
An illustration of the Tree-guided Transformer reasoning framework.

As shown in Figure 3, the Tree-guided Transformer accepts region-level visual features extracted via RoI alignment as guidance inputs into a multi-round prompting sequence. Each round corresponds to one level in the taxonomy tree, where the output from the previous level is concatenated as context input to the subsequent prompt, forming a progressive reasoning path. This process can be formalized as a generation tree $T=G_{1},G_{2},...,G_{n}$, where $G_{i}$ denotes the generation node at level *i*, and the final output path is $\hat{y}=y_{1},y_{2},...,y_{n}$. At each generation step $t_{i}$, the optimal predicted class is computed as

(8) $${\hat{y}}_{i}=\arg\max_{y\in C_{i}}P\left(y|x,{\hat{y}}_{<i}\right),$$

where $C_{i}$ denotes the candidate label set at level *i*, *x* represents the visual guidance feature, and ${\hat{y}}_{<i}$ is the prefix of the previously generated path. To improve, a self-consistency decoding strategy is incorporated, where the most probable output paths are selected and cross-validated. Additionally, a small number of annotated examples are employed to facilitate few-shot prompting, thereby enhancing the semantic accuracy and robustness of the generated label paths.

The LLaVA model is adopted as the backbone LLM, configured with an embedding dimension of 768 and 12 transformer layers. A shareable key–value cache mechanism is employed to store intermediate results of different prompts, reducing computational overhead across multi-round generation. Prompts are encoded using a structured template: “A target has been detected. Please identify its category at the {taxonomy level} from the candidates: $C_{i}$,” where $C_{i}$ is the candidate class set at that level. To further reinforce structural supervision, a hierarchical loss function, TreePathLoss, is introduced. The consistency term is defined as

(9) $$L_{tree}=\sum_{i=1}^{n}I\left[{\hat{y}}_{i}\neq y_{i}\right]\cdot \delta \left(i\right),$$

where δ(i) assigns higher weights to lower levels of the tree to emphasize the importance of fine-grained classification. This loss formulation penalizes inconsistent hierarchical paths and mitigates error propagation caused by incorrect upper-level predictions.

This module offers significant advantages for multi-object ecological recognition in agricultural settings. The hierarchical constraint improves the model’s discriminative power for visually similar classes, particularly low-frequency predator categories. The step-wise prompt structure aligns with human ecological cognition, enhancing interpretability. Moreover, the fusion of regional visual features and structured taxonomy-based prompts facilitates cross-modal reasoning, supporting accurate and explainable recognition of complex ecological imagery. The Tree-guided Transformer thus integrates seamlessly with the visual backbone and co-occurrence modeling modules to form a comprehensive solution for hierarchical recognition and ecological relationship inference.

#### 3.3.3. Knowledge-Augmented Transformer

The proposed Knowledge-Augmented Transformer was designed to embed agricultural ecological KGs into the visual backbone network, enabling the perception and modeling of semantic relationships among multiple objects in images. This module was constructed based on the Swin Transformer architecture, consisting of four stages. Each stage employed a hierarchical sliding window mechanism and cross-window connections to progressively extract semantic information from local to global scales. The feature map resolutions for Stages 1 to 4 were 56×56, 28×28, 14×14, and 7×7, respectively, with corresponding channel dimensions C=96, 192, 384, 768. Each stage contained two Swin Block modules, with the number of attention heads set to 3, 6, 12, 24 and a fixed window size of 7×7.

To provide reliable prior knowledge for the cross-modal embedding module, we first constructed agricultural knowledge graphs (KGs) from multiple domain-specific resources. Specifically, we collected structured information from authoritative agricultural ontologies (e.g., plant taxonomy databases, pest–disease knowledge bases, and agronomic practice guidelines), together with curated triples extracted from agricultural literature and expert-annotated datasets. Entities E were defined to represent objects of interest in agricultural systems, including crop species, pests, diseases, soil types, and ecological factors, while relations R described their interactions, such as “causes,” “affects,” “co-occurs with,” or “belongs to.” To ensure coverage of long-tail cases, we applied relation extraction and named-entity recognition (NER) methods to supplement missing triples from unstructured text sources, followed by expert verification to guarantee correctness. The resulting KG was represented as a set of triples (h,r,t), where *h* and *t* denote head and tail entities and *r* denotes the relation type. Each triple was validated against agricultural guidelines and merged into a unified schema to maintain consistency across heterogeneous sources. To enrich the connectivity, we further performed KG completion using embedding-based link prediction models (e.g., TransE/RotatE), which allowed the inference of plausible but unobserved relations. After completion, the final KG was pruned to remove noisy or low-confidence edges, and the resulting structured context graph G=(E,R) was used as input for subsequent graph convolutional encoding.

**Figure 4:**
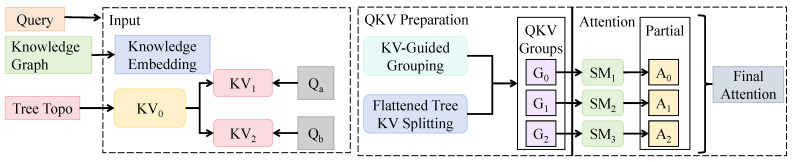
This figure illustrates the proposed Knowledge-Augmented Transformer architecture. The input undergoes QKV Preparation and is enriched with structural knowledge derived from the KG, including Tree Topology and knowledge embeddings. The key-value representations are grouped via the KV-Guided Grouping and Flattened Tree KV Splitting modules into semantic clusters (G0, G1, G2), which interact with query vectors (Qa, Qb) to generate local attention outputs (A0, A1, A2). These outputs are aggregated through Partial Attention and Final Attention modules to produce enhanced representations for ecological semantic modeling and target recognition.

To incorporate information from agricultural KGs, as shown in Figure 4, a cross-modal embedding module was designed. Structured context graphs G=(E,R) were constructed from entity nodes $e_{i}\in E$ and relational edges $r_{ij}\in R$. Each node was encoded as a vector $k_{i}$ and processed through a GCN model to produce knowledge embeddings $K_{i}$, which were then fused with visual features. A key-value guided attention mechanism was adopted for fusion: in each Swin Block attention module, the image feature $x_{i}$ was projected into a query vector $q_{i}=W_{q}x_{i}$, while the knowledge embedding was mapped to key and value vectors as $k_{i}=W_{k}K_{i}$ and $v_{i}=W_{v}K_{i}$. The attention weights were computed to update image features:

(10) $$\mathrm{Attention}\left(q_{i},k_{i},v_{i}\right)=\sum_{j=1}^{N}\frac{\exp(q_{i}^{⊤}k_{j}/\sqrt{d})}{\sum_{l=1}^{N}\exp\left(q_{i}^{⊤}k_{l}/\sqrt{d}\right)}v_{j}.$$

This formulation introduced structured prior knowledge into the attention mechanism, allowing the model to consider not only local textures but also semantic inter-object relationships such as “predator–pest” and “co-occurrence” during relevance computation.

A hierarchical knowledge-guided fusion mechanism was further incorporated. Based on the graph structure, entities were grouped into subgraphs (e.g., insect orders or predator families), each generating level-specific key-value mappings. In each attention module of the Swin layers, the corresponding level’s knowledge embeddings were selected for attention calculation, forming a hierarchical supervision mechanism expressed as

(11) $$f^{\left(l\right)}=\mathrm{Attn}\left(W_{q}^{\left(l\right)}x^{\left(l\right)},W_{k}^{\left(l\right)}K^{\left(l\right)},W_{v}^{\left(l\right)}K^{\left(l\right)}\right)+x^{\left(l\right)}.$$

Here, *l* denotes the layer index, $K^{\left(l\right)}$ refers to the knowledge embeddings selected for that level, and $x^{\left(l\right)}$ is the input feature at the current layer. Residual connections were used to enhance model stability. To improve semantic consistency, a knowledge-guided query offset was introduced as follows:

(12) $${\tilde{q}}_{i}=q_{i}+\mu \cdot \phi \left(K_{i}\right).$$

In this formulation, μ is a learnable parameter and ϕ(·) is the knowledge offset function. This design enables adaptive adjustment of the query vector based on the semantic graph, aligning the semantic and visual feature spaces.

This module and the Tree-guided Transformer module were jointly modeled through object detection results and graph entity anchors. Specifically, the class path predicted by the Tree-guided Transformer was mapped to a graph node $e_{i}$ and used as a conditioning signal for the Knowledge-Augmented Transformer. This reverse guidance path formed a conditionally enhanced cross-modal attention mechanism, mathematically expressed as

(13) $$\mathrm{FinalFeature}\left(x_{i}\right)=\mathrm{Transformer}\left(x_{i}|\mathrm{PromptTree}\left({\hat{y}}_{i}\right)\right).$$

Here, ${\hat{y}}_{i}$ is the class path output by the LLM, and the PromptTree function maps it to a graph-based condition. Through dual-path semantic and structural injection, the proposed framework significantly enhanced generalization, recognition accuracy, and semantic interpretability in ecological scenarios. It was especially effective in improving stability and semantic coherence in tasks involving misidentified predators, occluded multi-object detection, and co-occurrence recognition in complex farmland ecosystems.

#### 3.3.4. Ecological Semantic Alignment Loss

In multi-object recognition and ecological modeling tasks, conventional loss functions such as cross-entropy loss typically focus solely on classification accuracy while ignoring the underlying semantic hierarchy and ecological relations among targets. Such “flat” label assumptions are often inadequate for pest–predator co-occurrence recognition, where entities exhibit hierarchical taxonomic dependencies (e.g., “Hymenoptera–Braconidae–Trichogramma”) and ecological interactions (e.g., predation, co-habitation, competition). To address this, an Ecological Semantic Alignment Loss (ESA, denoted as $L_{ESA}$) was developed to enforce consistency between model outputs and ecological semantic structures during training.

This loss consists of three components: category classification loss, hierarchical path preservation loss, and semantic relation preservation loss. Let the ground-truth class path for target *i* be $Y_{i}=y_{i}^{1},y_{i}^{2},y_{i}^{3}$ and the predicted path be ${\hat{Y}}_{i}={\hat{y}}_{i}^{1},{\hat{y}}_{i}^{2},{\hat{y}}_{i}^{3}$, with corresponding graph embeddings $k(y_{i}^{l})$ and $k({\hat{y}}_{i}^{l})$. The semantic alignment loss is defined as

(14) $$L_{ESA}^{\left(i\right)}=\sum_{l=1}^{3}\left(\lambda_{1}\cdot \mathrm{CE}\left({\hat{y}}_{i}^{l},y_{i}^{l}\right)+\lambda_{2}\cdot {\left|k\left({\hat{y}}_{i}^{l}\right)-k\left(y_{i}^{l}\right)\right|}_{2}^{2}\right).$$

The first term represents standard cross-entropy loss, while the second enforces semantic embedding proximity in the graph space. Here, $\lambda_{1}$ and $\lambda_{2}$ are weighting coefficients. For co-occurring object pairs (i,j), ecological relations defined in the graph as $r_{ij}\in \mathrm{Predation},\;\text{Co-habitation},\;\mathrm{None}$ were encoded as vectors $r_{ij}^{*}$, with predicted relation ${\hat{r}}_{ij}$. A relation consistency loss was introduced as follows:

(15) $$L_{rel}^{\left(i,j\right)}=\lambda_{3}\cdot \left(1-\cos\left(\phi \left(\hat{r}*ij\right),\phi \left(r_{ij}^{*}\right)\right)\right).$$

Here, ϕ(·) is the relation embedding function and cos denotes cosine similarity. This term encourages the predicted inter-object relation to align semantically with the ecological KG. The overall loss is given by

(16) $$L_{total}=\frac{1}{N}\sum_{i=1}^{N}L_{ESA}^{\left(i\right)}+\frac{1}{M}\sum_{(i,j)\in P}L_{rel}^{\left(i,j\right)}.$$

Here, *N* is the number of targets, and *M* is the number of co-occurring target pairs in set P. Unlike traditional classification losses that focus solely on label accuracy, the proposed ESA loss enforces triple-level consistency among class, path, and inter-object relations in both semantic and structural spaces. This enhances model understanding of ecological multi-object scenes, as demonstrated in Algorithm 3.
**Algorithm 3** Computation of Ecological Semantic Alignment Loss  1:Initialize loss terms: $L_{cls}\leftarrow 0$, $L_{embed}\leftarrow 0$, $L_{rel}\leftarrow 0$  2:**for** i=1 to *N* **do**  3:     $L_{cls}\leftarrow L_{cls}+\mathrm{CrossEntropy}\left({\hat{Y}}_{i},Y_{i}\right)$  4:    **for** l=1 to 3 **do**           ▹ Each level in the hierarchical structure  5:         $L_{embed}\leftarrow L_{embed}+\mathrm{MSE}\left(k\left({\hat{y}}_{i}^{l}\right),k\left(y_{i}^{l}\right)\right)$  6:     **end for**  7:**end for**  8:**for all**(i,j)∈P**do**               ▹ Each co-occurring object pair  9:     $L_{rel}\leftarrow L_{rel}+\left(1-\cos\left(\phi \left({\hat{r}}_{ij}\right),\phi \left(r_{ij}^{*}\right)\right)\right)$10:**end for**11:$L_{total}\leftarrow \lambda_{1}\cdot L_{cls}+\lambda_{2}\cdot L_{embed}+\lambda_{3}\cdot L_{rel}$

From a mathematical perspective, this loss minimizes KL divergence via cross-entropy, Euclidean distance in the embedding space, and angular disparity via cosine similarity, forming a unified optimization framework for object recognition and relation modeling. This method is particularly suited for dense, co-occurrence-rich scenarios with high semantic redundancy, improving both detection performance and interpretability for rare targets such as predators.

## 4. Results and Discussion

### 4.1. Experimental Setup

#### 4.1.1. Data Splitting Protocol

To ensure fair evaluation and reproducibility, we adopted a stratified group splitting strategy. Images collected from the same field plot or acquisition session were treated as a group unit to prevent information leakage across subsets. The dataset was divided into 70% training, 10% validation, and 20% testing. For ablation and hyperparameter tuning, only the training and validation sets were used. In addition, we performed 5-fold stratified group cross-validation on the training+validation portion to reduce the bias introduced by a single partition. For each model, five independent runs with different random seeds were conducted, and the mean ± standard deviation (SD) of all evaluation metrics were reported. We further estimated 95% confidence intervals (CIs) of the metrics using bootstrap resampling on the test set. To assess whether the improvements over baselines are statistically significant, we applied paired *t*-tests. All splits, seeds, fold indices, and statistical testing protocols are fixed and will be released publicly to facilitate reproducibility.

#### 4.1.2. Hardware and Software Platform

The experiments were conducted on a system equipped with Ubuntu 22.04 LTS, an Intel Xeon Gold 6330 CPU (2.0 GHz, 64 cores), and an NVIDIA GeForce RTX 4090 GPU with 24 GB of memory. Data preprocessing was performed using Python 3.10 in combination with Albumentations and OpenCV. Model training and evaluation were implemented using the PyTorch 2.1.0 framework with CUDA 12.1 acceleration.

#### 4.1.3. Evaluation Metrics

To comprehensively evaluate the performance of the proposed model on pest–predator image recognition and ecological relationship modeling tasks, six metrics were adopted: precision, recall, F1-score, mAP at IoU threshold 0.5 (mAP@50), H-Acc, and co-occurrence accuracy (C-Acc). Among these, the first four are conventional metrics widely used in object detection and classification, while the latter two specifically assess the model’s ability to reason over hierarchical taxonomies and capture ecological co-occurrence relations. The mathematical definitions of these metrics are provided below:

(17) $$\mathrm{Precision}=\frac{TP}{TP+FP},\;\mathrm{Recall}=\frac{TP}{TP+FN},\;\text{F1-score}=\frac{2\cdot \mathrm{Precision}\cdot \mathrm{Recall}}{\mathrm{Precision}+\mathrm{Recall}},$$

(18) $$\mathrm{mAP}\text{@}50=\frac{1}{N}\sum_{i=1}^{N}{\mathrm{AP}}_{i}^{\mathrm{IoU}\geq 0.5},\;\mathrm{IoU}=\frac{\mathrm{Area}(B_{p}\cap B_{gt})}{\mathrm{Area}(B_{p}\cup B_{gt})},$$

(19) $$\text{H-Acc}=\frac{1}{N}\sum_{i=1}^{N}\delta \left(h_{i}={\hat{h}}_{i}\right),\;\text{C-Acc}=\frac{1}{N}\sum_{i=1}^{N}\delta \left(C_{i}={\hat{C}}_{i}\right).$$

Here, TP, FP, and FN represent true positives, false positives, and false negatives, respectively. $B_{p}$ and $B_{gt}$ denote the predicted and ground-truth bounding boxes. The intersection-over-union (IoU) criterion is employed to determine whether a predicted box matches the ground truth, defined as the ratio between the area of overlap and the area of union of $B_{p}$ and $B_{gt}$. Following common practice in object detection benchmarks such as PASCAL VOC [27], a prediction is counted as correct when IoU≥0.5, which ensures a reasonable trade-off between localization precision and robustness to annotation variability. *N* is the total number of samples, and ${\mathrm{AP}}_{i}^{\mathrm{IoU}\geq 0.5}$ denotes the average precision for the *i*-th class under this matching criterion. For hierarchical reasoning, $h_{i}$ and ${\hat{h}}_{i}$ denote the ground-truth and predicted hierarchical label paths, respectively, and we adopt the hierarchical accuracy (H-Acc) metric [28], which measures the proportion of correctly predicted nodes along the taxonomic path from root to leaf, rather than the harmonic accuracy used in other domains. For ecological relationship modeling, $C_{i}$ and ${\hat{C}}_{i}$ indicate the true and predicted pest–predator co-occurrence pairs, and the indicator function δ(·) returns 1 if the condition is satisfied and 0 otherwise. Together, these metrics comprehensively evaluate the model’s performance, not only in terms of detection accuracy but also its ability to capture hierarchical semantic structures and ecological co-occurrence relations, thereby providing a rigorous basis for quantitative analysis.

#### 4.1.4. Baseline

To assess the performance of the proposed pest–predator recognition framework, comparisons were conducted across multiple task dimensions using state-of-the-art baseline models covering image classification, multi-object detection, hierarchical reasoning, and knowledge-enhanced modeling.

For image classification, five representative architectures were selected: ViT [29], ResNet-50 [30], Swin-Tiny [31], ConvNeXt-T [32], and EfficientNet-B3 [33]. ViT introduced the pure Transformer structure into image classification, excelling in global context modeling. ResNet-50, a classical residual network, remains a strong CNN baseline due to its stable convergence. Swin-Tiny incorporates hierarchical local windows for enhanced locality and scalability. ConvNeXt-T aligns modern CNN design with Transformer performance, achieving a balance between efficiency and accuracy. EfficientNet-B3 utilizes compound scaling and neural architecture search to deliver high parameter efficiency and strong generalization. These models offer a diverse spectrum of depth, granularity, and efficiency for evaluating adaptability to agricultural image classification tasks.

In multi-object detection, five detection frameworks were employed: YOLOv5 [34], Faster R-CNN [35], YOLOv8 [36], DETR [37], and RTMDet [38]. YOLOv5 is a widely deployed real-time detector balancing accuracy and speed. Faster R-CNN represents the two-stage detection paradigm with strong region proposal and localization capacity. YOLOv8, the latest generation of the YOLO series, excels in precision and small object detection. DETR introduces a Transformer-based end-to-end detection pipeline that eliminates the need for non-maximum suppression and hand-crafted anchors. RTMDet, a recent anchor-free lightweight detector, is optimized for high-density, multi-scale object detection in field imagery. These models collectively span CNN-based and Transformer-based paradigms, reflecting technological trends and deployment feasibility for pest detection tasks.

For hierarchical reasoning, five models were adopted: Tree-LSTM [39], H-Softmax [40], Hierarchical Attention Network (HAN) [41], HiGCN (Hierarchical Graph Convolutional Network) [42], and H-TDNN (Hierarchical Top-Down Neural Network) [43]. Tree-LSTM employs tree-structured RNNs to capture label dependencies. H-Softmax reduces computational cost in large-class settings through hierarchical factorization. HAN leverages attention at multiple granularity levels for document modeling. HiGCN uses graph convolution to propagate and embed label hierarchies, suitable for ecological tree structures. H-TDNN simulates top-down semantic inference paths, aligning with human cognitive logic. Together, these models form a rigorous benchmark for evaluating structural reasoning capabilities in ecological taxonomy.

For knowledge-enhanced modeling, five multimodal methods were included: UNITER [44], ClipCap [45], BLIP [46], ERNIE-ViL [47], and K-Adapter [48]. These models incorporate external knowledge, language prompts, or semantic priors to enhance vision-language alignment. ClipCap learns image-to-language generation via contrastive pretraining. BLIP constructs a joint embedding space through large-scale vision-language pairs. ERNIE-ViL enhances deep semantic understanding via knowledge-injected representations. K-Adapter integrates plug-in knowledge modules into language models for controllable expression. These methods serve as strong references for evaluating the proposed co-occurrence modeling and knowledge-aware reasoning modules.

### 4.2. Performance Comparison of Image Classification Models

This experiment aims to validate the effectiveness of the proposed pest–predator recognition method in the image classification task, with a particular focus on evaluating key metrics such as precision, recall, F1-score, and hierarchical classification accuracy. From the perspective of holistic image-level recognition, a systematic comparison was conducted against five representative image classification models, encompassing diverse paradigms including self-attention architectures (ViT), classical convolutional backbones (ResNet-50), hierarchical window-based attention mechanisms (Swin-Tiny), modernized convolutional networks (ConvNeXt-T), and parameter-efficient models (EfficientNet-B3).

**Figure 5:**
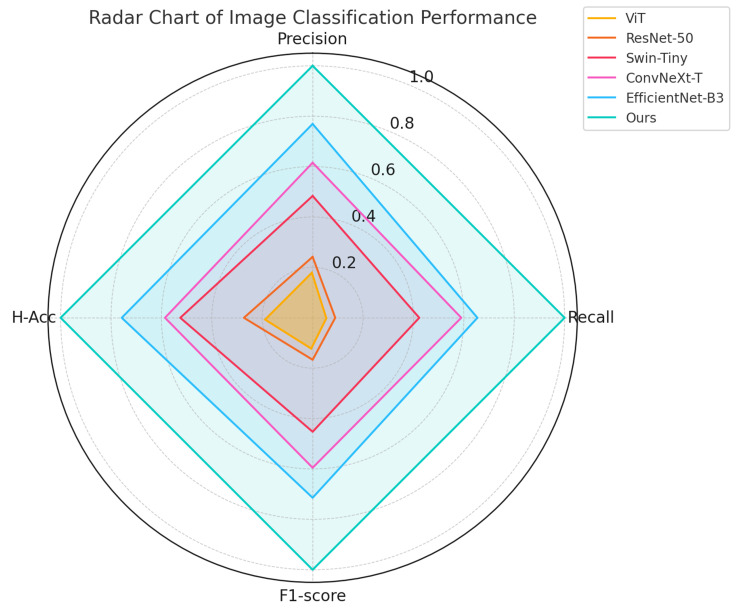
Normalized radar chart of image classification performance across four metrics.

As shown in Table 2 and Figure 5, the proposed method achieved the highest scores across all four evaluation metrics, with an F1-score of 88.5% and a hierarchical classification accuracy of 82.3%, outperforming the best-performing baseline by more than 2.4%. From a theoretical perspective, the structural differences among models directly influenced their effectiveness in this task; while ViT offers global modeling capabilities, its lack of inductive bias toward local structures leads to suboptimal performance in identifying fine-grained objects in complex agricultural scenes. ResNet-50 constructs deep representations via stacked convolutions, demonstrating strong feature extraction but limited inter-class discrimination. Swin-Tiny enhances local perception through hierarchical shifted windows, which is well-suited for dense object regions in farmland images. ConvNeXt-T maintains the benefits of convolution while integrating layer normalization and GELU activation from Transformers, thereby improving classification robustness and generalization. EfficientNet-B3 leverages compound scaling to optimize parameter efficiency and model capacity. In contrast, the proposed method integrates a structured prompt-guided mechanism and knowledge-enhanced modules, thereby enhancing semantic differentiation and hierarchical reasoning. Furthermore, an ecological semantic alignment loss is incorporated during training to compensate for limitations in conventional classifiers regarding hierarchical inference, leading to superior performance in recognizing complex ecological structures.

### 4.3. Performance Comparison of Object Detection Models

This experiment was designed to assess the detection performance of the proposed method in multi-object recognition and co-occurrence modeling of farmland pests and predators, in comparison with several state-of-the-art object detection frameworks. The selected baselines cover a wide range of detector families, including two-stage detectors (Faster R-CNN), real-time YOLO series (YOLOv5/YOLOv8), Transformer-based architectures (DETR), and anchor-free lightweight detectors (RTMDet). Evaluation metrics include precision, recall, mAP@50, and co-occurrence recognition accuracy (C-Acc), comprehensively reflecting performance in multi-scale object detection and ecological relationship modeling.

**Figure 6:**
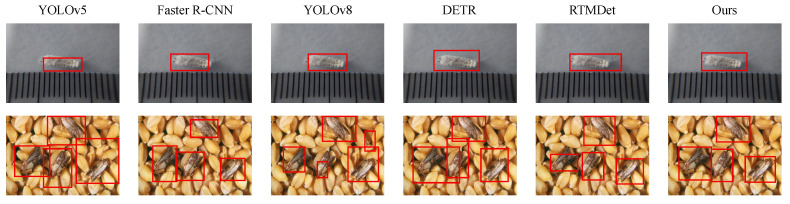
Visualization of detection results by different methods.

**Figure 7:**
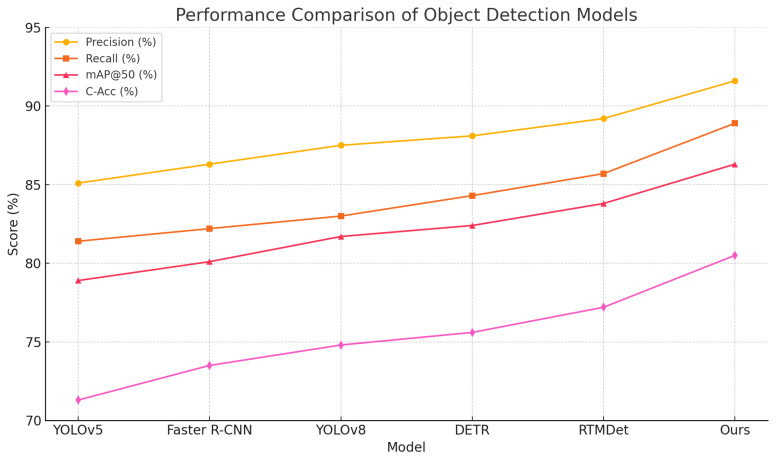
Line chart comparing the performance of object detection models across multiple metrics.

As presented in Table 3, Figure 6 and Figure 7, the proposed method outperformed all baselines across the four evaluation metrics, achieving an mAP@50 of 86.3% and a C-Acc of 80.5%, significantly surpassing the best-performing baseline, RTMDet. These results demonstrate the superior localization and co-occurrence awareness capabilities of the proposed method in complex ecological imagery. From a theoretical standpoint, differences in architectural design and underlying mathematical mechanisms explain the performance variations. YOLOv5, as a lightweight framework with static anchors and feature pyramids, offers efficiency but struggles with overlapping objects and cluttered backgrounds. Faster R-CNN benefits from region proposals for higher precision but suffers from multi-stage bottlenecks and slower inference. YOLOv8 incorporates refined backbones and detection heads to improve small-object detection while maintaining speed. DETR utilizes global self-attention for structural context modeling, yet its positional encoding and convergence remain challenging. RTMDet eliminates anchor boxes and refines decoding, enhancing detection in dense scenes. However, most of these models lack explicit modeling of ecological co-occurrence relationships and semantic structure constraints. The proposed method addresses these gaps by integrating a knowledge-augmented Transformer and co-occurrence attention mechanism, which reinforce inter-object ecological reasoning in the feature space. Additionally, prompt-guided class path prediction improves both spatial accuracy and semantic consistency, resulting in superior performance in ecological multi-object detection tasks.

### 4.4. Performance Comparison of Hierarchical Reasoning Models

This experiment was conducted to evaluate the structural understanding and semantic discrimination capabilities of different hierarchical reasoning models in the ecological classification of pests and predators in farmland scenarios. The evaluation focused on five metrics: precision, recall, F1-score, H-Acc, and C-Acc. The task simulated a three-level ecological classification tree for pests and natural enemies, with comparative methods including tree-structured recursive networks (Tree-LSTM), hierarchical softmax (H-Softmax), hierarchical attention networks (HAN), graph convolutional methods (HiGCN), and top-down reasoning networks (H-TDNN).

**Figure 8:**
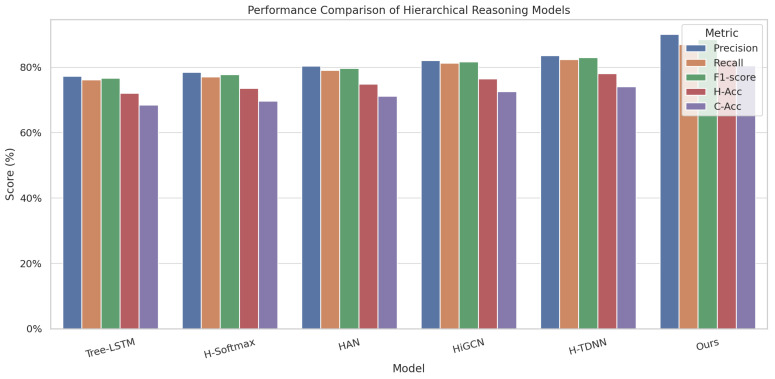
Bar chart comparing the performance of hierarchical reasoning models across multiple evaluation metrics.

As shown in Table 4 and Figure 8, the proposed method achieved superior results across all metrics, with an F1-score of 88.5% and an H-Acc of 82.3%, significantly outperforming the second-best H-TDNN (83.0%, 78.1%). These results demonstrate strong advantages in both semantic structural understanding and ecological category discrimination. From a modeling perspective, performance in hierarchical tasks is largely determined by the models’ capabilities in structural encoding, path reasoning, and semantic preservation. Tree-LSTM captures node dependencies via recursive structures but suffers from degraded information propagation in deep or unbalanced trees. H-Softmax reduces computation via probabilistic path decomposition, yet fails to account for semantic distances between paths. HAN enhances syntactic focus through multi-level attention but lacks adaptability to the nested semantics of label trees in image domains. HiGCN propagates label structure through graph convolutions, reinforcing inter-layer dependencies but lacking explicit path inference. H-TDNN mimics top-down semantic selection paths, offering better structural guidance but ignoring the dynamic influence of image content on path decisions. In contrast, the proposed method integrates a Tree-Guided Prompt mechanism with LLM, combining image region embeddings with structural priors for step-wise guided semantic generation. Moreover, a semantic alignment loss is employed to impose soft constraints between generated paths and KG structures, enabling accurate modeling of structural paths and image-text semantic linkage, thereby yielding significant improvements in hierarchical recognition and co-occurrence perception tasks.

### 4.5. Performance Comparison of Knowledge-Enhanced Models

This experiment aimed to compare the performance of various knowledge-enhanced models in the pest–predator image recognition task, with emphasis on recognition metrics (precision, recall, F1-score), C-Acc, and hierarchical understanding (H-Acc). In this task, knowledge-enhanced models serve as a bridge between visual representation and external ecological semantics by injecting KGs, prompts, or semantic embeddings to guide better understanding of inter-object relationships in multi-object recognition scenarios.

**Figure 9:**
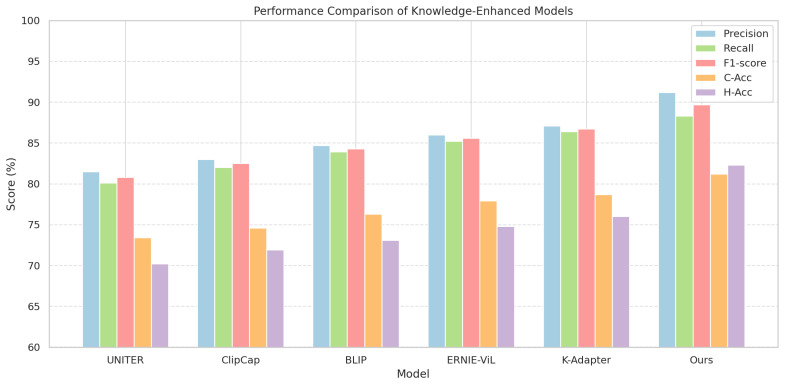
Performance comparison of various knowledge-enhanced models across five evaluation metrics.

As shown in Table 5 and Figure 9, the proposed method significantly outperformed representative models across all five metrics, achieving an F1-score of 89.7%, C-Acc of 81.2%, and H-Acc of 82.3%. These results highlight superior capabilities in both object recognition and ecological co-occurrence modeling. Structurally, knowledge injection mechanisms and semantic reasoning abilities varied substantially across models. UNITER adopts cross-modal pretraining for vision–language alignment but lacks explicit support for KGs. ClipCap combines image encoding with language generation for concept abstraction, yet lacks hierarchical structural constraints. BLIP builds a multimodal alignment space via image-text matching but struggles with structural relation modeling. ERNIE-ViL integrates knowledge embeddings into a vision transformer using pretrained language models, enhancing deep semantic reasoning, although it remains unoptimized for ecological structures. K-Adapter employs modular knowledge injection with stable expression but lacks explicit co-occurrence logic modeling from ecological KGs. In contrast, the proposed model incorporates KG embeddings, co-occurrence attention mechanisms, and prompt-guided path generation. Through interaction-aware attention between semantic entities and vision transformer features, a multi-head aggregation mapping is constructed between multimodal features and knowledge nodes on the computation graph, aligning semantic space with ecological knowledge structure. This mathematical path of image–text–knowledge fusion enables both high-accuracy recognition and interpretable ecological semantics in multi-object scenarios.

### 4.6. Discussion

#### 4.6.1. Analysis of Co-Occurrence Attention Mechanism

In the literature, a variety of attention mechanisms have been proposed, including channel attention, spatial attention, self-attention, co-attention, graph attention, multi-head attention, and scaled dot-product attention. Each type is designed to emphasize specific aspects of feature interactions. For instance, channel and spatial attention modules are well suited for emphasizing salient regions within single-modality visual features, while self-attention and multi-head attention are widely used for capturing long-range dependencies in sequential or grid-structured data. Graph attention excels in modeling structured relationships when the graph topology is explicitly known, and co-attention is often employed in cross-modal tasks such as vision–language alignment.

In contrast, the goal of our task is to model ecological co-occurrence relations between pests and predators under agricultural environments, where the interactions are neither purely spatial nor purely sequential but are inherently relational and context-dependent. Therefore, we adopt a co-occurrence attention mechanism that treats pest–predator pairs as candidate relational units and dynamically re-weights their contributions according to the contextual cues from both visual and knowledge representations. Compared with purely spatial or channel-based mechanisms, this design directly encodes ecological interactions rather than focusing only on local saliency; while self-attention and multi-head variants could in principle be applied, they would require learning relations in a fully data-driven manner without exploiting the structured co-occurrence priors, which may reduce interpretability and increase data requirements. Similarly, although graph attention networks (GATs) are powerful, they assume a fixed graph structure; in our setting, the co-occurrence graph is image-specific and dynamically changing, making direct GAT application less effective.

Thus, our choice of co-occurrence attention achieves a balance between relational expressiveness and computational efficiency while remaining closely aligned with the ecological semantics of the task. This design also ensures that the learned attention weights can be readily interpreted as strength indicators of pest–predator interactions, which is advantageous for ecological reasoning and practical agricultural decision-making.

#### 4.6.2. Analysis of Data Augmentation

Although a broad spectrum of image augmentation strategies has been explored in prior studies, ranging from photometric adjustments (e.g., gamma correction, color temperature modification) to advanced generative methods (e.g., GAN-based synthesis, style transfer, physics-based rendering), this work employed a focused set of augmentations that are directly aligned with the characteristics of agricultural field data. In particular, illumination perturbation, occlusion simulation, and local deformation were adopted, as they closely reflect environmental variability, partial target occlusion from vegetation or soil, and morphological diversity in insect postures frequently observed under real-world farmland conditions. These methods are computationally efficient, ecologically grounded, and effective in improving robustness without the need for additional annotations or synthetic data generation, while more sophisticated approaches such as generative modeling or physics-based rendering have the potential to simulate rare or complex scenarios, but they typically incur substantial computational cost, require domain-specific priors, and may introduce artifacts that reduce ecological validity. Similarly, certain low-level photometric transformations (e.g., gamma or color temperature shifts) were not prioritized, given that the collected dataset already exhibits sufficient variability in illumination. Overall, the selected augmentations represent a balance between ecological relevance, computational feasibility, and interpretability. A systematic comparison with more advanced augmentation techniques constitutes an important direction for future work.

#### 4.6.3. Analysis of Model Transferability and Robustness

From a theoretical perspective, the proposed framework incorporates several design choices that enhance its potential transferability to other domains and robustness to variations in shooting conditions. First, the integration of agricultural knowledge graphs provides a layer of domain-invariant prior knowledge: relationships among crops, pests, and ecological factors are relatively stable across ecosystems, which helps constrain the learning process and reduces overfitting to dataset-specific biases. Second, the adoption of tree-guided hierarchical reasoning ensures that predictions are structured according to taxonomic consistency, enabling more reliable generalization even when encountering unseen or rare species in new environments. Third, the data augmentation strategies—such as illumination perturbation, occlusion simulation, and morphological deformation—explicitly expose the model to variations commonly observed under different acquisition conditions, thereby improving its resilience to changes in lighting, background, or target appearance. Although these theoretical considerations indicate that the model is equipped with mechanisms to enhance cross-domain generalization, systematic empirical validation on independent datasets from other regions and under diverse acquisition conditions remains an essential direction for future work. Such evaluations will provide concrete evidence of the framework’s adaptability and further guide extensions toward broader ecological applications.

#### 4.6.4. Limitation and Future Work

Despite the notable performance improvements and promising application potential demonstrated by the proposed hierarchical pest–natural enemy recognition and co-occurrence modeling approach, several limitations remain. The construction of the current KG primarily relies on static modeling based on pre-existing agro-ecological datasets and expert knowledge, which limits the model’s adaptability to regional ecological differences. Future research will focus on the dynamic update and region-specific adaptation of the KG. One direction involves incorporating temporal data and multisource sensor inputs to build an ecological graph with a dynamic update mechanism, enabling the model to respond to seasonal changes, geographical variations, and emergent pest outbreaks.

## 5. Conclusions

This study addresses the core challenge of pest–natural enemy image recognition and ecological relationship modeling in agricultural fields. A tree-guided Transformer mechanism has been developed, and a vision transformer structure incorporating co-occurrence attention and KG embedding has been introduced. The model is jointly optimized across three dimensions: structural awareness, semantic understanding, and co-occurrence modeling. Experimental results demonstrate that the proposed method outperforms mainstream models across multiple metrics. In the image classification task, an F1-score of 88.5% and an H-Acc of 82.3% were achieved. In the object detection task, the method yielded a precision of 91.6%, an mAP@50 of 86.3%, and a C-Acc of 80.5%. In tasks involving hierarchical reasoning and knowledge enhancement, significant advantages were also observed, with both the F1-score and C-Acc exceeding 89%. These results indicate that the proposed model not only delivers strong recognition performance but also effectively captures ecological semantic relationships among targets, demonstrating excellent generalizability and interpretability.

## Figures and Tables

**Table 1:** Statistics of the pest–predator co-occurrence dataset.

Data Type	Sample Count	Source	Details
RGB images	12,000	Field + Open data	1920 × 1080 resolution
Infrared images	8500	Synchronized capture	640 × 480 resolution
Text descriptions	2400	GBIF, FAO, Atlases	Morphology + behavior
Instance annotations	48,000	Expert labeled	Bounding boxes
Co-occurrence pairs	13,000	Expert labeled	Predation, competition
Taxonomy labels	60	Integrated	3 levels: order–family-species

**Table 2:** Performance comparison of image classification models (mean ± SD over 5 runs). The 95% confidence intervals (CIs) are reported in brackets. *p*-values are from paired *t*-tests against the best baseline (EfficientNet-B3).

Model	Precision (%)	Recall (%)	F1-Score (%)	H-Acc (%)
ViT	81.3 ± 0.7 [80.0, 82.6]	78.9 ± 0.9 [77.1, 80.6]	80.1 ± 0.6 [78.9, 81.3]	72.4 ± 0.8 [70.8, 74.0]
ResNet-50	83.5 ± 0.5 [82.4, 84.6]	79.6 ± 0.7 [78.2, 80.9]	81.5 ± 0.6 [80.3, 82.7]	75.1 ± 0.7 [73.7, 76.6]
Swin-Tiny	85.7 ± 0.6 [84.5, 86.9]	82.2 ± 0.8 [80.6, 83.7]	83.9 ± 0.7 [82.5, 85.2]	77.6 ± 0.6 [76.4, 78.8]
ConvNeXt-T	86.9 ± 0.5 [85.8, 87.9]	83.5 ± 0.6 [82.3, 84.7]	85.1 ± 0.6 [84.0, 86.3]	78.2 ± 0.7 [76.8, 79.6]
EfficientNet-B3	88.3 ± 0.6 [87.0, 89.5]	84.0 ± 0.7 [82.6, 85.5]	86.1 ± 0.5 [85.0, 87.2]	79.9 ± 0.6 [78.7, 81.1]
**Ours**	**90.4 ± 0.5** [89.4, 91.4] **	**86.7 ± 0.6** [85.5, 87.9] **	**88.5 ± 0.4** [87.7, 89.3] **	**82.3 ± 0.5** [81.3, 83.3] *

* p<0.05, ** p<0.01 versus EfficientNet-B3 (paired *t*-test).

**Table 3:** Performance comparison of object detection models (mean ± SD over 5 runs). The 95% confidence intervals (CIs) are reported in brackets. *p*-values are from paired *t*-tests against the best baseline (RTMDet).

Model	Precision (%)	Recall (%)	mAP@50 (%)	C-Acc (%)
YOLOv5	85.1 ± 0.7 [83.8, 86.4]	81.4 ± 0.8 [79.8, 83.0]	78.9 ± 0.6 [77.7, 80.1]	71.3 ± 0.7 [69.9, 72.7]
Faster R-CNN	86.3 ± 0.6 [85.1, 87.5]	82.2 ± 0.7 [80.8, 83.6]	80.1 ± 0.5 [79.1, 81.2]	73.5 ± 0.6 [72.3, 74.7]
YOLOv8	87.5 ± 0.5 [86.5, 88.6]	83.0 ± 0.6 [81.8, 84.2]	81.7 ± 0.6 [80.5, 82.9]	74.8 ± 0.7 [73.4, 76.2]
DETR	88.1 ± 0.6 [87.0, 89.3]	84.3 ± 0.5 [83.3, 85.4]	82.4 ± 0.5 [81.3, 83.4]	75.6 ± 0.6 [74.4, 76.8]
RTMDet	89.2 ± 0.6 [88.1, 90.4]	85.7 ± 0.6 [84.5, 86.9]	83.8 ± 0.5 [82.8, 84.9]	77.2 ± 0.6 [76.0, 78.4]
**Ours**	**91.6 ± 0.5** [90.6, 92.6] **	**88.9 ± 0.6** [87.7, 90.1] **	**86.3 ± 0.4** [85.5, 87.1] **	**80.5 ± 0.5** [79.5, 81.5] *

* p<0.05, ** p<0.01 versus RTMDet (paired *t*-test).

**Table 4:** Performance comparison of hierarchical reasoning models (mean ± SD over 5 runs). The 95% confidence intervals (CIs) are reported in brackets. *p*-values are from paired *t*-tests against the best baseline (H-TDNN).

Model	Precision (%)	Recall (%)	F1-Score (%)	H-Acc (%)	C-Acc (%)
Tree-LSTM	77.3 ± 0.8 [75.8, 78.8]	76.2 ± 0.9 [74.5, 77.9]	76.7 ± 0.7 [75.4, 78.0]	72.1 ± 0.8 [70.6, 73.6]	68.5 ± 0.9 [66.8, 70.2]
H-Softmax	78.5 ± 0.7 [77.1, 79.9]	77.1 ± 0.8 [75.6, 78.7]	77.8 ± 0.7 [76.4, 79.2]	73.6 ± 0.7 [72.2, 75.0]	69.7 ± 0.8 [68.1, 71.3]
HAN	80.4 ± 0.6 [79.2, 81.6]	79.1 ± 0.7 [77.7, 80.5]	79.7 ± 0.6 [78.5, 80.9]	74.9 ± 0.6 [73.7, 76.1]	71.2 ± 0.7 [69.8, 72.6]
HiGCN	82.1 ± 0.6 [81.0, 83.3]	81.3 ± 0.7 [79.9, 82.7]	81.7 ± 0.6 [80.6, 82.9]	76.5 ± 0.6 [75.3, 77.7]	72.6 ± 0.7 [71.2, 74.0]
H-TDNN	83.6 ± 0.5 [82.6, 84.7]	82.4 ± 0.6 [81.2, 83.6]	83.0 ± 0.5 [82.0, 84.0]	78.1 ± 0.6 [76.9, 79.3]	74.1 ± 0.6 [72.9, 75.3]
**Ours**	**90.1 ± 0.5** [89.1, 91.1] **	**87.0 ± 0.6** [85.8, 88.2] **	**88.5 ± 0.5** [87.5, 89.5] **	**82.3 ± 0.5** [81.3, 83.3] **	**80.5 ± 0.5** [79.5, 81.5] **

** p<0.01 versus H-TDNN (paired *t*-test).

**Table 5:** Performance comparison of knowledge-enhanced models (mean ± SD over 5 runs). The 95% confidence intervals (CIs) are reported in brackets. *p*-values are from paired *t*-tests against the best baseline (K-Adapter).

Model	Precision (%)	Recall (%)	F1-Score (%)	C-Acc (%)	H-Acc (%)
UNITER	81.5 ± 0.7 [80.1, 82.9]	80.1 ± 0.8 [78.5, 81.7]	80.8 ± 0.6 [79.6, 82.0]	73.4 ± 0.7 [72.0, 74.8]	70.2 ± 0.8 [68.6, 71.8]
ClipCap	83.0 ± 0.6 [81.8, 84.2]	82.0 ± 0.7 [80.6, 83.4]	82.5 ± 0.6 [81.3, 83.7]	74.6 ± 0.6 [73.4, 75.8]	71.9 ± 0.7 [70.5, 73.3]
BLIP	84.7 ± 0.6 [83.5, 85.9]	83.9 ± 0.6 [82.7, 85.1]	84.3 ± 0.5 [83.3, 85.4]	76.3 ± 0.6 [75.1, 77.5]	73.1 ± 0.7 [71.7, 74.5]
ERNIE-ViL	86.0 ± 0.5 [85.0, 87.0]	85.2 ± 0.6 [84.0, 86.4]	85.6 ± 0.5 [84.6, 86.6]	77.9 ± 0.6 [76.7, 79.1]	74.8 ± 0.6 [73.6, 76.0]
K-Adapter	87.1 ± 0.5 [86.1, 88.1]	86.4 ± 0.5 [85.4, 87.4]	86.7 ± 0.5 [85.7, 87.7]	78.7 ± 0.5 [77.7, 79.7]	76.0 ± 0.6 [74.8, 77.2]
**Ours**	**91.2 ± 0.5** [90.2, 92.2] **	**88.3 ± 0.6** [87.1, 89.5] **	**89.7 ± 0.5** [88.7, 90.7] **	**81.2 ± 0.5** [80.2, 82.2] **	**82.3 ± 0.5** [81.3, 83.3] **

** p<0.01 versus K-Adapter (paired *t*-test).

## Data Availability

The data presented in this study are available on request from the corresponding author.
